# Mandatory health insurance for the informal sector in Tanzania—has it worked anywhere!

**DOI:** 10.3389/frhs.2023.1247301

**Published:** 2023-10-02

**Authors:** Amani Thomas Mori

**Affiliations:** ^1^ Bergen Center for Ethics and Priority Setting, Department of Global Public Health and Primary Care, University of Bergen, Bergen, Norway; ^2^ Department of Development Studies, Muhimbili University of Health and Allied Sciences, Dar es Salaam, Tanzania; ^3^ National Institute of Medical Research, Muhimbili Research Center, Dar es Salaam, Tanzania

**Keywords:** Tanzania, informal sector, health, insurance, universal health coverage

## Abstract

Many countries in sub-Saharan Africa are struggling to expand voluntary health insurance schemes to raise finances toward achieving universal health coverage. With more than three-quarters of the population without any insurance, the government of Tanzania has unsuccessfully tried to pass a Bill proposing a mandatory, nationwide scheme to cover the large and diverse informal sector. The Bill proposed an annual premium of ∼150 USD for a household of six or 65 USD per person. Studies in Tanzania and Kenya have shown that the majority of people in the informal sector are unwilling and unable to pay premiums as low as 4 USD, mostly due to poverty. Mandatory health insurance for the informal sector is not common in this region, mostly because it is difficult to enforce. Successful insurance schemes have included significant subsidies from tax revenues. Tanzania should not seek to raise funds for health through an unenforceable insurance scheme but rather should consider a largely tax-funded scheme for the informal sector. Contributions through low-cost voluntary schemes can enhance social contracts, reduce out-of-pocket expenditure, and promote efficient utilization. In addition, progressive health taxes should be imposed on harmful products (tobacco, alcohol, sugary drinks, etc.) to raise more funds while addressing the increasing burden of non-communicable diseases. Furthermore, efficiency in the use of scarce health resources should be promoted through realistic prioritization of public services, the use of Health Technology Assessment, and strategic purchasing.

## 1. Introduction

Universal Health Coverage (UHC) as enshrined in the Sustainable Development Goals aims to ensure that everyone has access to the healthcare they need without suffering financial hardship (1). However, more than half of sub-Saharan African countries, including Tanzania, rely heavily on out-of-pocket (OOP) payments, estimated to exceed 30% of total health expenditure (THE) (2). A recent review showed that out of 36 countries in this region, only Ghana, Rwanda, Gabon, and Burundi have insurance coverage of above 20% (3). High OOP payments may expose households to catastrophic and impoverishing expenses (4). The incidence of catastrophic health expenditure i.e., health payments that exceed 10% of THE in this region is estimated at 16.5% (5). Even small expenses can cause financial hardship because of the extreme poverty prevalent in this region and will displace other crucial household investments. Households often turn to selling assets, using savings, taking on loans, or forgoing other necessities to cope with such health financial shock (4, 6, 7), which keeps them in a vicious cycle of poverty (4).

Tanzania is a lower-middle-income country, with a population of 61.3 million people. Since independence, Tanzania has gone through several phases of health reforms. Following independence, Tanzania issued the Arusha Declaration in 1967 with its policy of Socialism and Self Reliance, which included the abolition of user fees in public health facilities (8). These reforms also aimed to ensure universal access to social services and to reverse the rural-urban dichotomy by passing budgets that prioritized rural and marginalized communities and focus on preventive services. In 1977, the government banned private for-profit medical practice and took on the ambitious task of providing health services for free through taxation. Following the economic crisis in the 1980s and the Structural Adjustment Programs, more reforms were implemented in the early 1990s that lifted the ban on private for-profit providers and introduced (in phases) user fees in public health facilities (9).

### 1.1. Health insurance for universal health coverage

In 1999, Tanzania started the National Health Insurance Fund (NHIF), followed by the Community Health Fund (CHF) in 2001. NHIF is a mandatory scheme, primarily for formal employees, with a contribution of 6% shared equally between the employee and the employer. NHIF's revenues come from member contributions (85.9%) and returns on investment (13.5%) (10). In contrast, CHF is a voluntary scheme for the informal sector mostly in rural areas, where around 70% of the population lives. Weak management, poor understanding of the concept of risk pooling, poor quality of services in public facilities, a benefits package that restricted members to only access outpatient health services (11–13), and inability and unwillingness to pay annual premiums of between 5,000 and 10,000 TZS (∼2–4 USD) per household of six (11, 13, 14) were the main challenges behind low enrollment rates. This was despite the presence of exemptions and waiver mechanisms for the poor. In 2016, CHF was reformed to an “improved Community Health Fund” (iCHF), with a flat annual premium of 30,000 TZS (US$ 13), and an improved benefits package including referral to regional level in-patient services [in Dar es Salaam the premium was higher at 150,000 TZS (65 US$)] (15).

Insurance coverage in Tanzania has remained low over the last two decades of implementation. Only about 15% of Tanzanians (8% through NHIF), equivalent to 9.1 million had health insurance by the end of 2021 (16). This represents a significant decrease compared to the 32% coverage reported in 2018, of which 8% were under NHIF, 21% under iCHF, and 3% under private schemes (17). Overall, health insurance contributes 12% of THE in Tanzania, while OOP payments, government, and donors contribute 34%, 24%, and 32%, respectively (18). Since 2016, government and donor contribution to THE has been decreasing while OOP has been increasing (18), thus shifting the financing burden toward the patients.

In 2022, the government proposed mandatory health insurance as a strategy to cover the large informal sector and raise additional revenue for health. The Bill for the establishment of mandatory Universal Health Insurance (UHI) and Tanzania Insurance Regulatory Agency (TIRA) was however withdrawn from the parliament twice. One of the TIRA's roles was to determine those who cannot pay so that they can be exempted. The Bill also proposed the incorporation of the iCHF into the new scheme. In both instances, the UHI Bill was withdrawn to allow more consultation with the parliamentary committee and other stakeholders (19). The contentious issue was the proposed annual premium of 350,000 TZS (∼150 USD) for a household of six or 150,000 TZS (∼65 USD) per person—and the lack of a clear definition of entitlements to the beneficiaries. To enhance enrolment, the Bill proposed that all citizens must present evidence of having health insurance when seeking a driving license, motor vehicle insurance, or admitting children to advanced secondary school, or higher education institutions (20). These enforcement strategies would further marginalize the poor who are unlikely to own motor vehicles or require driving licenses or admission to higher learning institutions.

The level of poverty particularly in rural areas, where most poor, vulnerable, and the informal sector people live further cast doubts on whether mandatory contributions are feasible. A recent financial survey of 2023 showed that more than 80% of adult Tanzanians receive seasonal or occasional income (21). The most recent Household Budget Survey also reported that about 33% of the people in rural areas and 15% in urban, live in poverty and could not afford their basic needs. Their monthly consumption expenditures were 362,000 TZS and 535,000 TZS, respectively (22). Interestingly, about half of the household consumption (46.1%) was held by the highest 20% income group and only 3.1% by the lowest 10% income group. The proposed UHI premium represents about 10% and 5% of the household consumption in rural and urban areas, respectively–but does not cater to significant inequity in the distribution of income and consumption across the informal employment sector. This level of poverty explains why rural residents were unable and unwilling to pay the annual premiums of 5,000–10,000 TZS required for iCHF. It is therefore unimaginable that the proposed premium of 350,000 TZS will be affordable to rural residents. Furthermore, no plans have been made and explained to improve access to quality health care or to cross-subsidize for those less able to pay and to exempt the poorest. A recent study in rural Tanzania has shown that poor households utilize outpatient and inpatient care much less compared to the well-off, regardless of their iCHF enrollment status (23). The authors invited studies to explore the reasons behind this inequity. However, one can speculate the lack of literacy to navigate the healthcare system, bureaucracy in healthcare provision or transport-related and other indirect costs associated with care-seeking could play an important role.

## 2. Discussion

### 2.1. Have mandatory health insurance schemes for the informal sector worked elsewhere in Africa?

Mandatory health insurance for the informal sector is not common in Africa. The reason being the informal sector involves low and often irregular incomes, hence it is difficult to join prepayment schemes. The degree of informality means the government has little insight or capacity to assess and tax income. Even if informal workers enroll, the attrition rate is usually very high. A recent review showed that most mandatory insurance schemes target formal sector employees and not the informal sector. Even when the mandatory contributions extend to the informal sector they remain voluntary (24). For example in Ghana, “*NHIS is mandatory but because the informal sector has to make premium payment before they are enrolled, the authorities are unable to enforce the mandatory nature of the scheme*” (25). To accommodate this, a large proportion of the population of Ghana is exempt and many district health insurance schemes (DHISs) charge relatively low annual premiums ranging between US$ 2 and 4 (26). Despite the low levels of premiums, coverage has never exceeded 60%. In Kenya, the National Hospital Insurance Fund has been mandatory for the formal and informal sector since 1998 (27), and the premium is US$4 per month (500 Kenya Shilling), yet studies have shown that it is unaffordable to the majority i.e., 60% (7) and enrollment among informal sector citizens is very low.

One major challenge with mandatory health insurance contribution in the informal sector is how to enforce the law. In Rwanda, the insurance coverage in the informal sector is above 80%. However, enrolment in the *Mutuelles de Santé*/Community-Based Health Insurance schemes (CBHIs) is not only mandatory by law but local government officials are held responsible to increase enrollment rates and can enforce penalties on those who do not pay. These penalties may include monetary fines, confiscation of livestock, banning entrance to local markets, or denying administrative documents to those without insurance cards (28, 29). The population of Tanzania is more than four times that of Rwanda and more importantly, it is spread over a geographical area that is 36 times bigger. Such a large population and lack of financial and human resources coupled with a more complex five-tier health system spread over such a huge area will make enforcing similar penalties in Tanzania very challenging.

### 2.2. Policy recommendations

#### 2.2.1. The tax-funded insurance scheme is more sustainable

Public financing is dubbed as the surest and most sustainable means to finance health to achieve UHC (30, 31). Gabani et al. (32), using data from 124 countries showed that transitions from OOP-dominant to public-financed systems improved life expectancy, reduced under-five mortality, and the incidence of CHE more than transitions to social health insurance systems. The paper cited limited coverage of health insurance and high implementation costs as the reasons behind their finding (32). Other studies have also shown that all countries that have managed to expand coverage of health services to the poor did so through general government revenues (33). Unaffordability and unwillingness to pay premiums are well-documented among the reasons for the poor performance of voluntary schemes in Tanzania and other developing countries (11, 13, 14, 34), hence tax-based contributions to any financing strategy are largely unavoidable (3, 24, 35–38). The Ghanaian NHIS with coverage of 58%, gets 70% of its revenues from the national health insurance levy of 2.5%, which is a value-added tax imposed on goods and services (39). Finances raised through formal and informal sector premiums account for less than 5% of THE (39). Nigeria has also passed a mandatory health insurance law but included a tax-based non-contributory financing mechanism to cover the poor, vulnerable, and informal sectors that represent more than 70% of the population i.e., 190 million (40). In Ethiopia there is wide coverage of CBHIs in rural areas, however, contributions account for less than 1% of THE (41). In Rwanda, member contributions account for two-thirds of the CBHIs revenues but the poorest are fully subsidized through tax revenues or donor contributions (42).

#### 2.2.2. Introduce ring-fenced health taxes

Taxation of products or goods that are deemed harmful to individuals and costly to society (health taxes) such as tobacco, alcohol, gambling, and sugary beverages is increasingly becoming important in developing countries, not only to discourage unhealthy consumption but also to increase domestic revenues for health (43). In the case of Thailand, which introduced UHC in 2002, a 2% levy on alcohol and tobacco generated 50–60 million USD annually for health financing (44). Ghana recently passed a law to introduce health taxes and there has been a call to ensure these taxes are ring-fenced for health. Otherwise, it has been argued that the intended target of reducing OOP health expenditure will not be realized (45). Tanzania should follow suit by imposing health taxes to raise revenues and protect public health, which in return may reduce health expenses due to Non-Communicable Diseases.

#### 2.2.3. Enhancing efficient and equitable use of scarce resources

The World Health Report of 2010-“Health Systems Financing: The Path to Universal Coverage” indicated that up to 40% of health sources are wasted. Hence, in addition to trying to enhance the pool of pre-paid health contributions—it is critical that resources are well spent in the public interest. Establishing benefits packages to determine entitlements under public systems as well as the use of Health Technology Assessment (HTA) can enhance effective prioritization and targeting of resource use to services with the highest benefit and equity-inducing impact (31). In 2014, WHO urged member states to establish national HTA systems, to systematically inform policy decisions, including priority-setting, selection, procurement supply system management and use of health interventions and/or technologies, as well as the formulation of sustainable financing benefit packages, medicines, benefits management including pharmaceutical formularies, clinical practice guidelines, and protocols for public health programs (46). Yet studies have shown that Tanzania lacks a rigorous system to inform reimbursement decisions (47). Despite Tanzanian commitment to UHC, little progress has been made in HTA institutionalization despite a lot of efforts by international supporters (48). The quest to increase funding must go hand in hand with improved capabilities to establish priorities, improve purchasing arrangements, and ensure enhanced access to quality services for all Tanzanians.

#### 2.2.4. Involve the public in the discussion about health system reforms and expanding public communication and awareness

Public perception and preferences can determine the success or failure of health sector reforms. Therefore, extensive public involvement and support are crucial elements in creating a sustainable health financing system. What I observed with the failed Bill for UHI was complete neglect of public involvement in the discussions about the proposed health insurance reform, which eventually led to its rejection. There are various strategies that the public can be involved. First, for financing reforms that were going to impact such a large population, eliciting their willingness to pay through well-established methods such as contingent valuation was necessary. For example, in the Gambia, a household survey was conducted to determine the Willingness-to-Pay amount for the NHIF, which was 23.3 USD (49). Previous studies in Tanzania have reported 5 USD as the amount residents working in the informal sector were willing-to-Pay (14). It remains unknown whether these data were used to inform the premium for the proposed Bill. Second, public debates through different platforms were necessary to inform the bottom-up process rather than the top-down approach that was used. Although government officials and guests from the public held several live discussions about the UHI on television, these were largely polarized. On one side, government officials argued in favor of the proposed premium, and on the other side the public opposed it, some even called for free healthcare for all. One could observe a communication gap between policymakers and the public. Hence, better communication was imperative to enhance public understanding and prepare them for the reforms.

## 3. Conclusion

Mandatory health insurance for the informal sector is not easy to implement in Africa, and where it has worked the contribution, particularly among the poor has been small. About one-third of the people in rural and one-fifth in urban Tanzania live in poverty, hence are unlikely to afford large contributions to enroll in mandatory health insurance schemes. Therefore, the government should not seek to raise funds for health through an unenforceable insurance scheme but rather opt for a tax-funded scheme to cover the informal sector. Progressive health taxes should be imposed on harmful products (tobacco, alcohol, sugary drinks, etc.) to raise more funds while addressing the increasing burden of non-communicable diseases. Furthermore, efficiency in the use of scarce health resources should be promoted through realistic prioritization of public services, Health Technology Assessment, and strategic purchasing.

## Data Availability

The original contributions presented in the study are included in the article/Supplementary Material, further inquiries can be directed to the corresponding author.
